# Comparative study of young-old and old-old people using functional evaluation, gait characteristics, and cardiopulmonary metabolic energy consumption

**DOI:** 10.1186/s12877-023-04088-6

**Published:** 2023-06-29

**Authors:** Eunhye Chung, Su-Hyun Lee, Hwang-Jae Lee, Yun-Hee Kim

**Affiliations:** 1 Department of Medical Device Management & Research, SAIHST, Sungkyunkwan University, Seoul, 06351 Republic of Korea; 2 Department of Physical and Rehabilitation Medicine, Sungkyunkwan University School of Medicine, Suwon, 16419 Republic of Korea; 3 Robot Business Team, Samsung Electronics, Suwon, 16677 Republic of Korea; 4 Haeundae Sharing and Happiness Hospital, Pusan, 48101 Republic of Korea

**Keywords:** Aging, Gait characteristics, Cardiopulmonary metabolic energy consumption

## Abstract

**Background:**

Walking is an important factor in daily life. Among older adults, gait function declines with age. In contrast to the many studies revealing gait differences between young adults and older adults, few studies have further divided older adults into groups. The purpose of this study was to subdivide an older adult population by age to identify age-related differences in functional evaluation, gait characteristics and cardiopulmonary metabolic energy consumption while walking.

**Methods:**

This was a cross-sectional study of 62 old adult participants who were classified into two age groups of 31 participants each as follows: young-old (65–74 years) and old-old (75–84 years) group. Physical functions, activities of daily living, mood state, cognitive function, quality of life, and fall efficacy were evaluated using the Short Physical Performance Battery (SPPB), Four-square Step Test (FSST), Timed Up and Go Test (TUG), Korean Version of the Modified Barthel Index, Geriatric Depression Scale (GDS), Korean Mini-mental State Examination, EuroQol-5 Dimensions (EQ-5D) questionnaire, and the Korean version of the Fall Efficacy Scale. A three-dimensional motion capture system (Kestrel Digital RealTime System®; Motion Analysis Corporation, Santa Rosa, CA, USA) and two force plates (TF-4060-B; Tec Gihan, Kyoto, Japan) were used to investigate spatiotemporal gait parameters (velocity, cadence, stride length, stride width, step length, single support, stance phase, and swing phase), kinematic variables (hip, knee, and ankle joint angles), and kinetic variables (hip, knee, and ankle joint moment and power) of gait. A portable cardiopulmonary metabolic system (K5; Cosmed, Rome, Italy) was used to measure cardiopulmonary energy consumption.

**Results:**

The old-old group showed significantly lower SPPB, FSST, TUG, GDS-SF, and EQ-5D scores (*p* < 0.05). Among spatiotemporal gait parameters, velocity, stride length, and step length were significantly lower in the old-old group than in the young-old group (*p* < 0.05). Among the kinematic variables, the knee joint flexion angles during initial contact and terminal swing phase were significantly higher in the old-old than the young-old group (*P *< 0.05). The old-old group also showed a significantly lower ankle joint plantarflexion angle during the pre- and initial swing phases (*P* < 0.05). Among the kinetic variables, the hip joint flexion moment and knee joint absorption power in the pre-swing phase were significantly lower in the old-old than the young-old group (*P *< 0.05).

**Conclusion:**

This study demonstrated that participants 75–84 years of age had less functional gaits than their young-old counterparts (65–74 years old). As the walking pace of old-old people diminishes, driving strength to move ahead and pressure on the knee joint also tend to decrease together with stride length. These differences in gait characteristics according to age among older adults could improve our understanding of how aging causes variations in gait that increase the risk of falls. Older adults of different ages may require customized intervention plans, such as gait training methods, to prevent age-related falls.

**Trial registration:**

Clinical trials registration information: ClinicalTrials.gov Identifier: NCT04723927 (26/01/2021).

**Supplementary Information:**

The online version contains supplementary material available at 10.1186/s12877-023-04088-6.

## Background

The size of the global older adult population is rapidly increasing [1]. The current age at which a person is considered old is 65 years or older. In Japan, which has the world's largest aging population, researchers have called for ‘senior’ to be redefined as age 75 or older [2]. Previous research has shown that age-related body function changes such as slowing of movement and grip weakening occur 5 to 10 years later in life than they did 10 to 20 years ago, resulting in a ‘rejuvenation’ phenomenon [3]. Furthermore, people aged 65–74 years have better mental and physical health than previous generations at that age range, and they engage in more social activities than people older than 75 years. Some geriatric studies have compared the characteristics of people aged 65–74 years (young-old) with those of people aged 75–84 years (old-old) [4, 5]. For example, compared with young-old individuals, old-old people showed increased depression [6] and decreased health-promoting behavior and self-esteem [7]. In physical health, old-old people show pronounced decreases in gait function, such as increased muscle weakness in the lower extremities and increased gait disturbances, compared with young-old people [8, 9].

Among studies dealing with changes in older adults due to aging, gait studies are important because they can identify or predict clinical abnormalities related to aging [10]. In gait research, functional evaluation plays an important role in patient treatment planning and prognosis prediction [11]. Furthermore, kinematic and kinetic variables provide important information on the underlying causes of the gait impairment and can help guide treatment and intervention strategies among older adults [12]. In addition, cardiopulmonary metabolic energy efficiency testing is important for evaluating human exercise capacity and predicting disease outcomes [13], and research has confirmed that older people use energy less efficiently than younger adults during walking [14]. As such, functional assessment, gait analysis, and cardiopulmonary metabolic energy efficiency testing are important measures of mobility quality and functional capacity in older adults [15, 16]. Several previous studies have shown variations in function [17, 18], gait patterns [19–23], and age-related cardiopulmonary metabolic efficiency [24–26] between healthy older adults and younger adults. However, few studies have divided older adult participants into two age groups and evaluated function, gait, and cardiopulmonary metabolic energy use in a laboratory setting.

The purpose of this study was to investigate differences in gait measures among participants divided into young-old (65–74 years) and old-old groups (75–84 years) within a single laboratory environment. Results are intended to be used as strategic data for creating elderly-specific interventions to prevent the risks associated with aging, such as falls. Three main hypotheses of this study are: 1) physical performance assessments and participant-reported outcomes differ between the young-old and old-old groups; 2) spatiotemporal gait parameters, kinematics, and kinetics differ between the young-old and old-old groups; 3) cardiopulmonary metabolic energy efficiency differ between the young-old and old-old groups.

## Materials and methods

### Participants

This cross-sectional study included 62 older adult people without a history of neurological or psychiatric complaints ranging in age from 65–84 years (mean age, 74.16 ± 4.26 years; 29 males). They were split into two groups based on their ages, as follows: young-old (65–74 years; mean age, 70.6 ± 2.4 years; *n *= 31; 12 males) and old-old (75–84 years; mean age, 77.74 ± 2.07 years; *n* = 31; 17 males). Participants were excluded if they (1) experienced difficulty walking on their own due to issues such as visual field loss, (2) had severe dizziness that might cause a fall, or (3) demonstrated severe cognitive decline with a score of <  = 10 points on the Korean Mini-mental State Exam (K-MMSE) [27]. Samsung Medical Center's 'IRB number: 2020–09-172' Institutional Review Board approved this study protocol after receiving the informed consent of all subjects.

### Experimental protocol

**Fig. 1:**
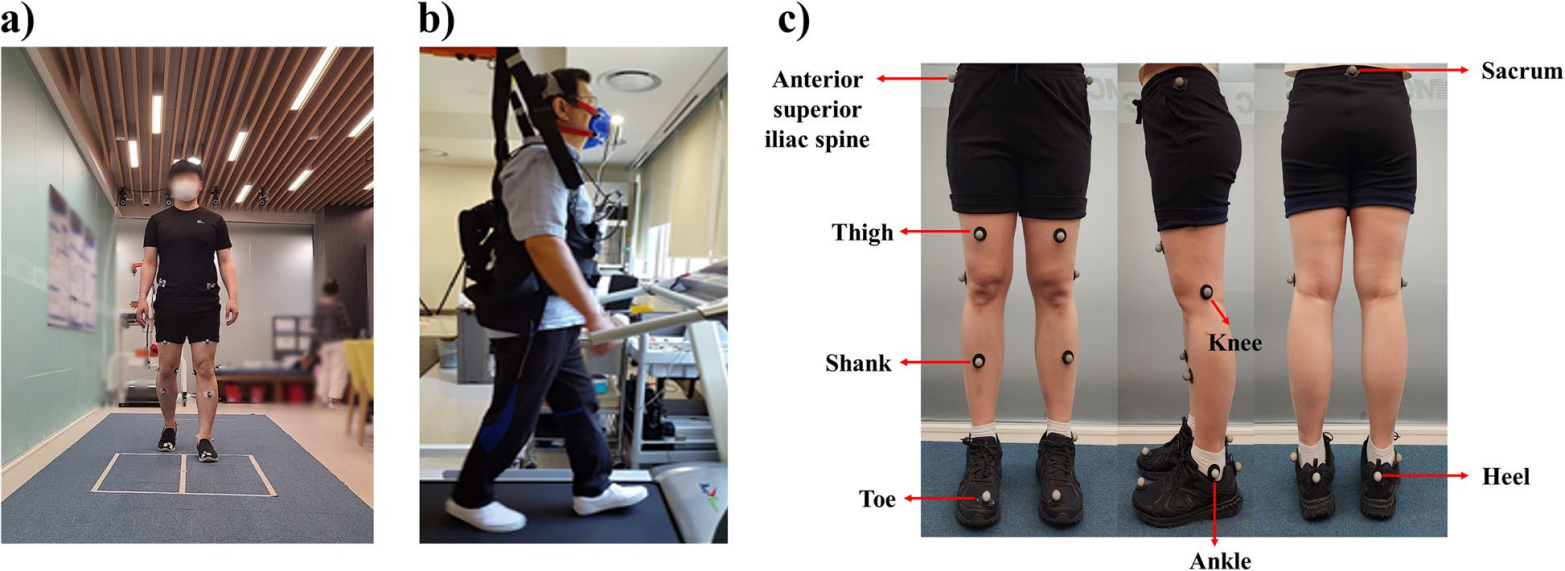
**a** Participant gait function was measured with a 3D motion capture system on a 10-m walkway at their preferred walking speed. **b** Participants cardiopulmonary metabolic energy consumption was measured on a treadmill at preferred walking speed. **c** Measured marker position

**Fig. 2:**
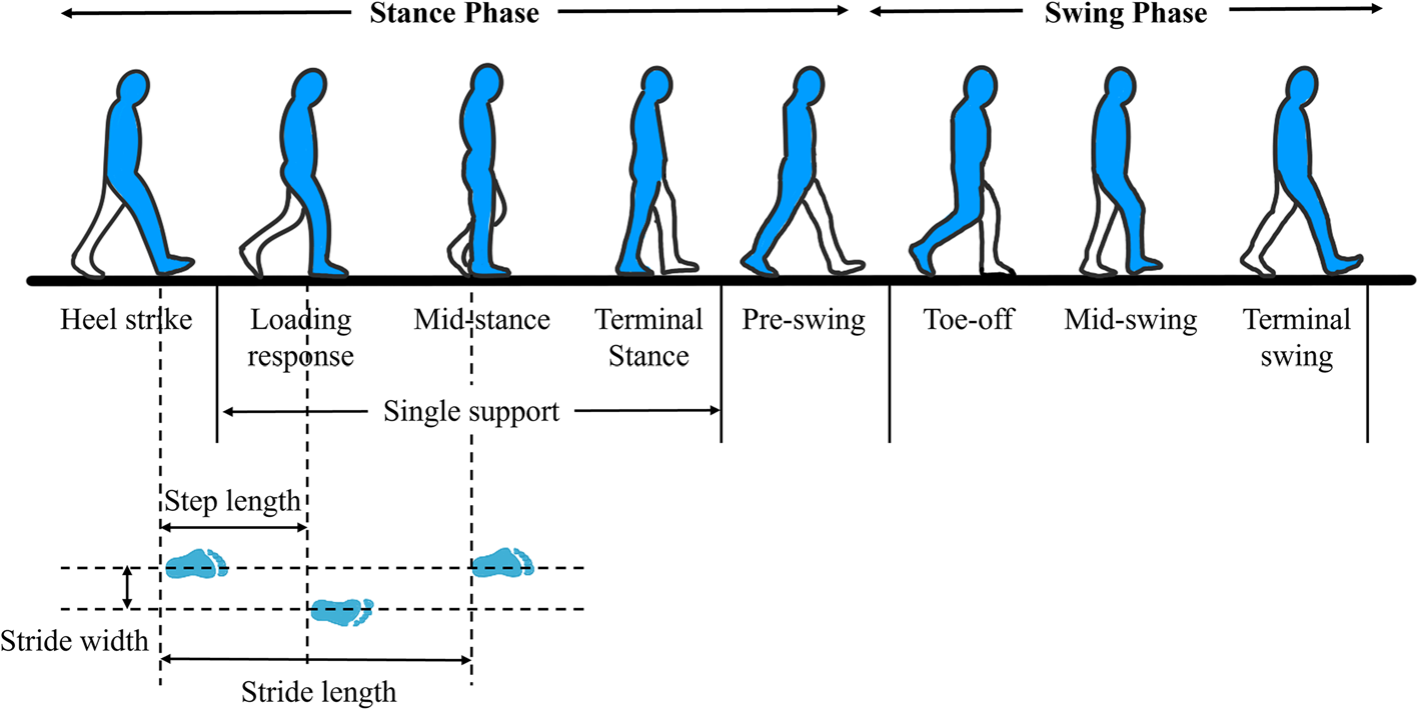
Phases of the normal gait cycle and spatiotemporal gait parameters

All study participants who consented to participate provided their sociodemographic information (age, sex, and educational attainment), height, weight, body mass index, and medical history (Table 1). Functional assessments were used to assess the differences of the participant's physical performance assessments and participant-reported outcomes. A three-dimensional (3D) motion capture system (Kestrel Digital RealTime System®; Motion Analysis Corporation, Santa Rosa, CA, USA) and two force plates (TF-4060-B; Tec Gihan, Kyoto, Japan) were used to investigate spatiotemporal, kinematic, and kinetic aspects of gait. The participants were asked to walk along a 10-m walkway at their preferred walking speed (Fig. 1). The spatiotemporal gait parameter variables were velocity (cm/s), cadence (step/min), stride length (cm), step length (cm), single support (% cycle), stance phase (% cycle), and swing phase (% cycle) [28] (Fig. 2). Gait kinematic variables were the hip, knee, and ankle joint angles (degree) in the sagittal plane during walking. Kinetic variables were sagittal plane, the hip, knee, and ankle joint moments (N·m/kg·m) and power (W/kg·m). Gait data were obtained subjectively through a walkway test in a motion analysis laboratory. While the participants were attached to a portable cardiopulmonary metabolic system (K5; Cosmed, Rome, Italy), baseline values were determined by measuring cardiopulmonary metabolic energy consumption data while standing comfortably for three minutes. Then, their metabolic rate was measured as they walked on a treadmill for six minutes at preferred walking speed. Three variables measured when evaluating cardiopulmonary metabolic consumption: The net cardiopulmonary metabolic energy cost (net VO2) is the energy expended by the body while walking a certain distance [29]. The energy expenditure measurement (EEm) is the total energy cost of maintaining constant conditions in the body plus the energy cost of physical activities [30]. The metabolic equivalent (MET) is an objective measure of the ratio between the rate at which a person expends energy while performing some specific physical activity and the mass of that person compared to a reference that is set by convention at 3.5 mL of oxygen per kilogram per minute, which is roughly equivalent to the energy expended when sitting quietly [31].

**Table 1:** Demographic characteristics of the young-old and old-old groups

	All participants	Young-old	Old-old	*P* value
Age [years]	74.16 (4.24)	70.58 (2.41)	77.74 (2.07)	< 0.001***
Sex [male:female]	(29:33)	(12:19)	(17:14)	0.309
Height [cm]	160.37 (7.67)	161.08 (6.40)	159.66 (8.81)	0.350
Weight [kg]	60.10 (7.92)	59.87 (7.42)	60.33 (8.50)	0.816
BMI [kg/m^2^]	23.39 (2.83)	23.07 (2.62)	23.71 (3.04)	0.822
Education [years]	10.44 (4.55)	11.16 (4.15)	9.73 (4.87)	0.384
Medical history, n [%]				
Neck pain	4 (6%)	2 (3%)	2 (3%)	1.000
Low back pain	6 (10%)	2 (3%)	4 (6%)	0.671
Knee osteoarthritis	8 (13%)	5 (8%)	3 (5%)	0.707
Rheumatoid arthritis	1 (2%)	0 (0%)	1 (2%)	1.000
High blood pressure	33 (53%)	13 (21%)	20 (32%)	0.126
Heart disease	5 (8%)	1 (2%)	4 (6%)	0.354

Continuous values are presented as mean (standard deviation). Values are presented as number (%). Young-old, 65–74 years; Old-old, 75–84 years

*Abbreviations:* *BMI* Body Mass Index

^*^Indicates a statistically significant defference between the young-old and old-old groups using paired t-test. ***P < 0.001

### Measurements

The physical performance assessments were as follows. The Short Physical Performance Battery (SPPB) [32] evaluates balance, lower limb muscle strength, and mobility by assessing three tasks: balancing, performing five sit-to-stand cycles, and walking at a normal pace. Each task is rated on a scale from 0 (worst) to 4 (best), and the overall summary score ranges from 0 (worst performers) to 12 (best performers), providing an objective measure of an individual's physical performance. The Four-Square Step Test (FSST) [33] evaluates dynamic stability and coordination by measuring the time it takes for a subject to step clockwise, then counterclockwise, through each quadrant. Subjects are instructed to face forward during the entire sequence, if possible. The Timed Up and Go Test (TUG) [34] assesses balance and functional exercise ability to predict the risk of falling. To assess TUG, participants are seated on a chair positioned against a wall, after which they are directed to rise from the chair, walk along a 3-m pathway at their normal pace, turn around, come back, and sit down once again. The timing of the task begins with the command "go" and concludes when the participant sits down again. The questionnaires used for participant-reported outcomes were as follows. The K-MMSE is a quick way to evaluate cognitive function and the participant's current state, with scores ranging from 0 to 30. A score of 24 points or more is considered 'normal', a score of 20 to 23 is 'suspected dementia', and a score of 19 points or less is 'definite dementia'. The Korean-Modified Barthel Index (K-MBI) [35] assesses the degree of independence in daily life through 10 items which has a five level scoring system according to the degree of help and direct observation/interview. The degree of dependence is evaluated on a scale from complete independence (100 points) to complete dependence (0 points). A score of 24 points or less is considered 'total dependence', a score of 25 to 49 is 'severe dependence', a score of 50 to 74 is 'moderate dependence', a score of 75 to 90 is 'mild dependence', and a score of 91 points or more is 'minimal dependence'. The Fall Efficacy Scale-Korea (FES-K) [36] assesses a subject's self-confidence in performing daily activities without falling. Participants express their fear of performing 10 activities on a scale from 1 point being ‘not at all confident’ and 10 points being ‘very confident’, with lower scores indicating greater fear of falling. The Geriatric Depression.

Scale Short Form (GDS-SF) [37] is an effective screening instrument for monitoring mood status and evaluating depression, which includes 15 yes/no questions. A score of 0 to 5 is 'normal’, a score 6 to 9 suggests 'depression', and a score 10 or more is almost always indicative of depression. The EuroQol-5 Dimension (EQ-5D) [38] consists of five domains, mobility (M), self-care (SC), usual activities (UA), pain/discomfort (PD), and anxiety/depression (AD), and each domain is scored as follows: no problem = level 1, some/moderate problems = level 2, extreme problem = level 3. According to the EQ-5D index: $1-(0.05+0.096\times \mathrm{M}2+0.418\times \mathrm{M}3+0.046\times \mathrm{SC}2+0.136\times \mathrm{SC}3+0.051\times \mathrm{UA}2+0.208\times \mathrm{UA}3+0.037\times \mathrm{PD}2+0.151\times \mathrm{PD}3+0.043\times \mathrm{AD}2+0.158\times \mathrm{AD}3+0.050\times \mathrm{N}3)$, if there is no problem in any of the five areas, the EQ-5D index = 0.95. When the EQ-5D index is divided into quintiles, 20% or less is 'very bad' and 20 to 40% is 'bad', 40 to 60% is 'average', 60 to 80% is 'good', and 80% or more is 'very good'. All assessments were performed by trained physical therapists blinded to the intervention task. A 3D motion capture system consisting of eight infrared cameras was used to measure spatiotemporal and kinematic data. Additionally, two force plates (TF-4060-B, Tec Gihan, Kyoto, Japan) embedded midway along the walkway were used to collect kinetic data. The Helen Hayes marker model was used to collect the trajectories of 15 markers placed on anatomical landmarks [39]. The motion capture system was able to define each marker during collection, allowing for real-time recording of marker position (Fig. 1). Markers were applied to the bilateral anterior superior iliac spine, sacrum, bilateral thigh, knee, shank, ankle, toe, and heel to enable 3D motion capture. Standing calibration was used to obtain a rotation matrix for each limb segment [40–42]. The Cosmed K5 wearable metabolic system was used to measure cardiopulmonary energy consumption. The Cosmed K5 portable cardiopulmonary metabolic system was placed on the upper body before respiratory metabolism measurement started, and each participant wore a face mask to ensure that breathing analysis was precise. This system works by using combined breath-by-breath technology to measure oxygen consumption (VO_2_, L/min) and carbon dioxide production (VCO_2_, L/min) and thereby evaluates physical performance to support clinical diagnoses. Specifically, it measures the flow, quantity, and volume of oxygen and carbon dioxide in exhaled breath. Broadly, the equipment senses the amount of respiration sent to it by a sample line attached to a turbine when the exhaled gas is discharged through the turbine. Sensors within the device analyze the data. Prior to each experiment, the flow turbine and gas analyzer of the Cosmed K5 analyzer unit were calibrated using a 3-L calibration syringe, gas, and regulator.

### Data processing and analysis

Data were automatically converted to 3D coordinates using CORTEX motion capture software version 5.5.0 (Motion Analysis Corporation, Santa Rosa, CA, USA), which has a sampling rate of up to 2000 Hz for cameras and up to 5000 Hz for force plates. A low-band pass filter was used to remove unnecessary noise and better observe the changes according to gait cycle. All data were calculated for each gait cycle using Ortho Track 6.6.4 software (Motion Analysis Corporation). The net cardiopulmonary metabolic energy costs during standing and walking were calculated using the Brockway equation as follows: $16.58\mathrm{VO}2 + 4.51\mathrm{VCO}2 - 5.90\mathrm{N}$ [43], which subtracts the mean data from the last minute of walking from the mean data from the last minute of baseline measurement [44]. EEm and METs were calculated from the average data in the last minute of walking.

### Statistical analysis

All statistical analyses were performed using SPSS version 22.0 (IBM Corporation, Armonk, NY, USA), and the significance level was set at 0.05. Results were calculated as the mean value with standard deviation. To determine the appropriate statistical tests to apply, we checked the distribution of the data for normality. Differences in demographic characteristics between the young-old and old-old were investigated using independent t-tests and chi-square tests. Significant variations in functional assessments, spatiotemporal gait parameters, kinematics and kinetics of gait, and cardiopulmonary metabolic energy efficiency were compared using paired t tests or the Mann–Whitney U test to determine statistically significant differences among groups.

## Results

### Differences in functional assessments between the young-old and old-old groups

Table 2 presents the functional differences observed between the two groups. The results show that the old-old group had significantly poorer gait and balance function than the young-old group on the SPPB, FSST, and TUG tests (*P* < 0.01). Moreover, the quality of life (EQ-5D) and mood state (GDS) of the old-old group were significantly lower than those of the young-old group (*P *< 0.05). However, there were no significant differences between the two groups in K-MMSE, K-MBI, and FES-K scores.

**Table 2:** Functional differences between the young-old and old-old groups

	Young-old	Old-old	*P* value
SPPB [total score: 12 points]	11.71 (0.59)	10.81 (1.28)	**0.001**^§§§^
FSST [sec]	7.59 (1.55)	8.90 (1.08)	0.001***
TUG [sec]	7.57 (1.00)	8.77 (1.35)	**0.001**^§§§^
K-MMSE [total score: 30 points]	26.77 (2.39)	26.00 (3.91)	0.743
K-MBI [total score: 100 points]	100.00 (0.00)	99.94 (0.36)	0.317
FES-K [total score: 100 points]	99.90 (0.54)	98.00 (6.98)	0.148
GDS-SF [total score: 15 points]	2.06 (3.08)	4.84 (4.48)	**0.002**^§§^
EQ-5D [total score: 1 point]	0.91 (0.08)	0.87 (0.09)	**0.021**^§^

Values are presented as mean (standard deviation)

*Abbreviations:* *SPPB* Short Physical Performance Battery, *FSST* Four-Square Step Test, *TUG* Timed Up and Go, *K-MBI* Korean version of the Modified Barthel Index, *GDS-SF* Geriatric Depression Scale Short Form, *K-MMSE* Korean Mini-Mental State Examination, *EQ-5D* EuroQol-5 Dimensions, *FES-K* Fall Efficacy Scale-Korea

^*^Indicates a statistically significant difference between the young-old and old-old groups using paired t-test. ***P < 0.001

^§^Indicates a statistically significant difference between the young-old and old-old groups using Mann–Whitney U test. ^§^*P* < 0.05, ^§^^§^*P* < 0.01, ^§^^§^^§^*P* < 0.001

### Gait characteristics and differences between older adult groups

**Fig. 3:**
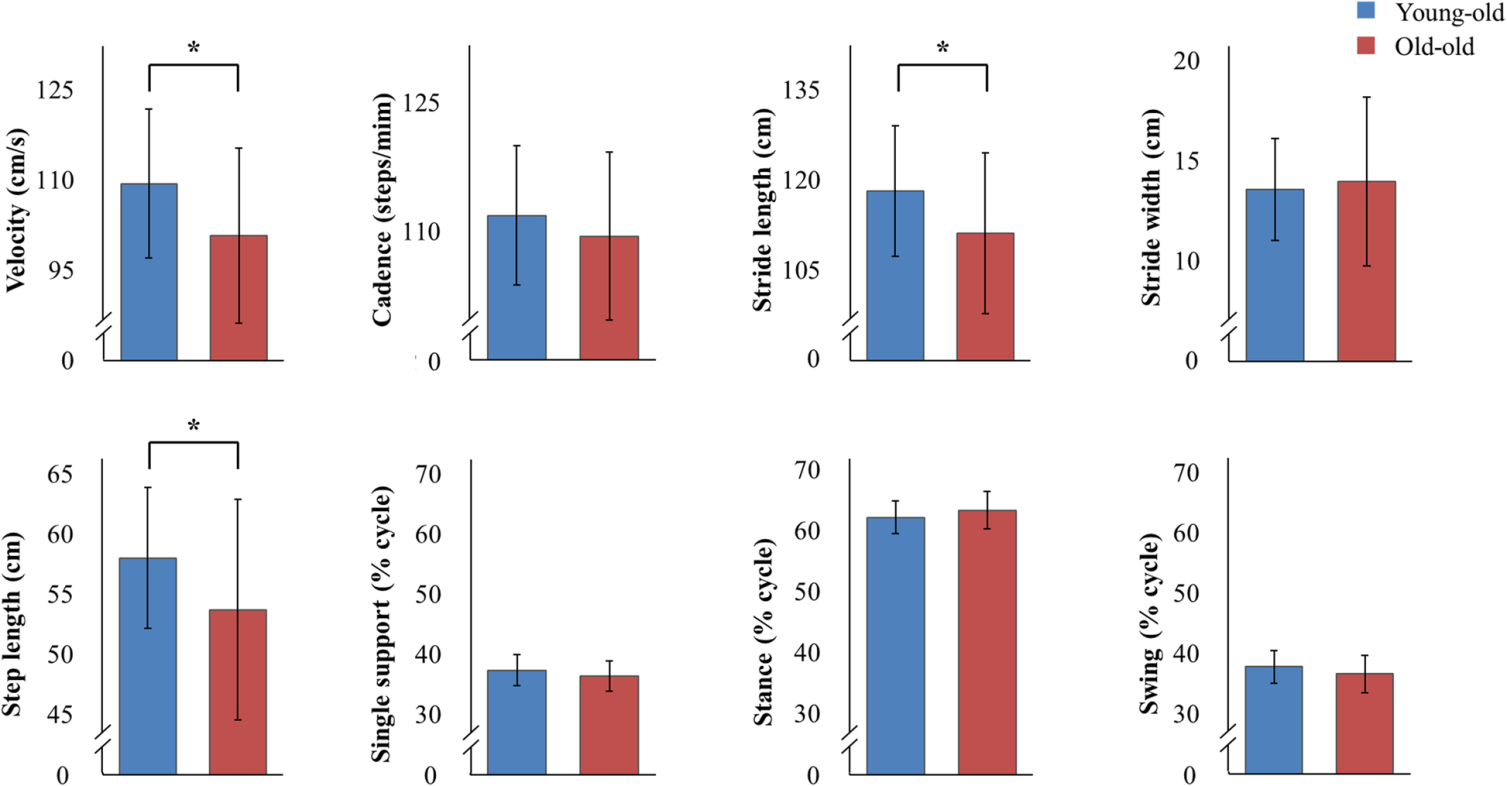
Comparison of spatiotemporal gait parameters between the young-old and old-old groups. *Significant difference between groups on paired t-test (*P* < 0.05)

**Fig. 4:**
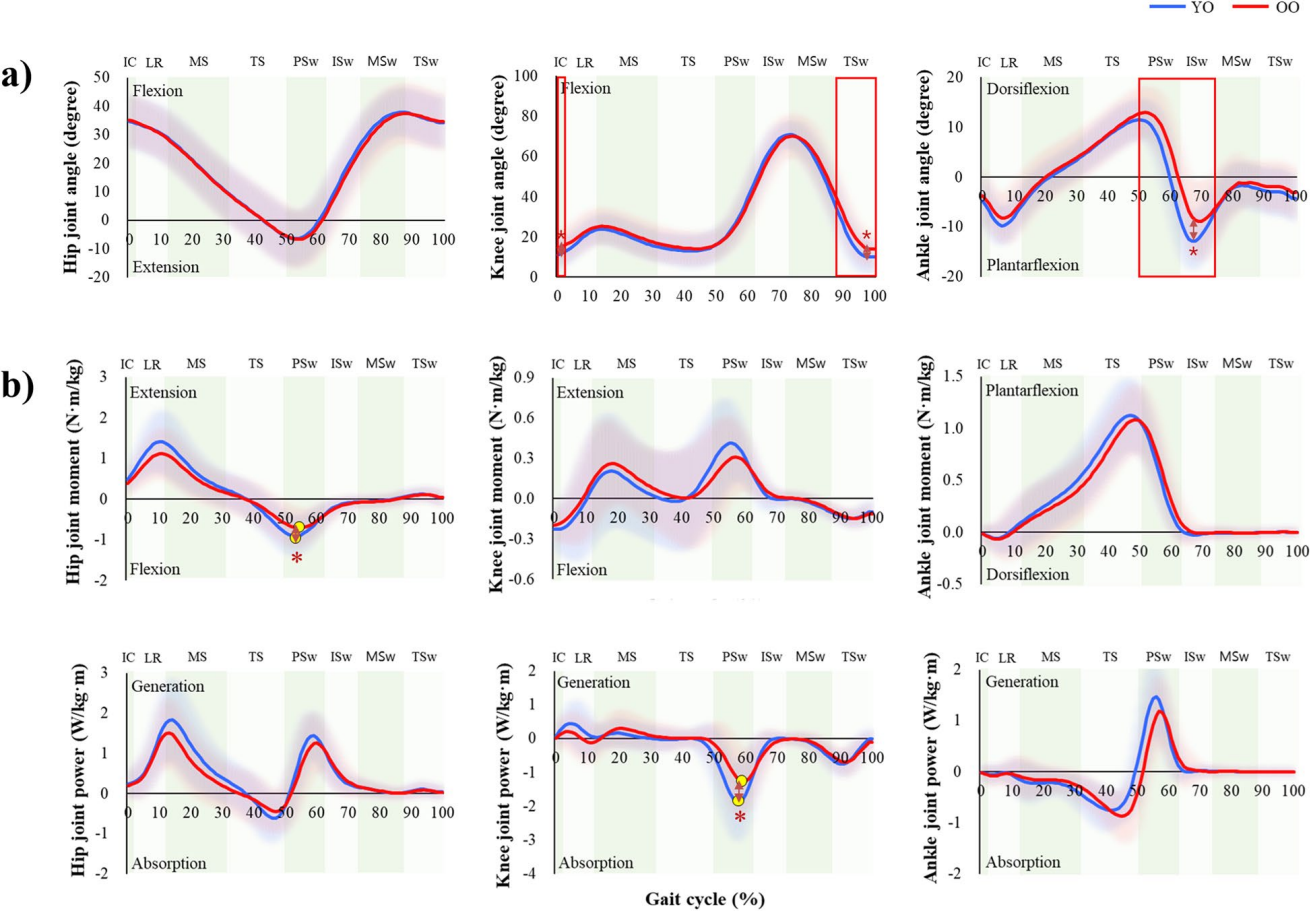
**a** Joint angles over a gait cycle in the young-old and old-old groups, which differed in knee joint angle during the initial contact and terminal swing phases and ankle joint plantarflexion during the pre- and initial swing phases. **b** Peak joint moments and power over a gait cycle in the young-old and old-old groups, which differed significantly in hip joint flexion moment and knee joint absorption power in the pre-swing phase. Red rectangular lines represent significant differences (paired t-test, *P* < 0.05) between groups. Yellow circles represent significant differences (paired t-test, *P* < 0.05) between groups

Gait characteristics based on spatiotemporal gait parameters were compared and analyzed between the young-old and old-old groups. Results showed that the old-old group had significantly poorer gait function than the young-old group in terms of velocity, stride length, and step length (*P *< 0.05) (Fig. 3). The groups did not differ in their cadence, stride width, single support, stance, or swing. The results of the comparative analysis of kinematic gait characteristics between groups can be seen in Fig. 4. The hip joint angle during the gait cycle did not differ significantly between groups, but the knee and ankle joint angle were significantly different (Supplementary Table S1). The knee joint angle was more flexed during the initial contact and terminal swing phases in the old-old group than in the young-old group (*P *< 0.05). The ankle joint angle was less plantarflexed during the pre- and initial-swing phases of the gait cycle in the old-old group than in the young-old group (*P* < 0.01). The results of comparative analysis of kinetic moment and power gait characteristics of the two groups can be seen in Fig. 4, Supplementary Table S2, and Supplementary Table S3. In the pre-swing phase, the hip joint flexion moment and knee joint absorption power were significantly different between groups. At the peak of the hip joint flexion moment in the pre-swing phase, the old-old group exhibited a significantly less flexed moment than the young-old group (*P *< 0.05). Knee and ankle moments did not differ significantly between groups. There was a difference between the young-old and old-old groups in peak knee joint power in the pre-swing phase; the old-old group used significantly less absorption power than the young-old group (*P *< 0.05). Hip and ankle power did not exhibit any substantial differences between groups. The peak ground reaction force during the gait cycle did not differ significantly between groups (Supplementary Fig. S1).

### Cardiopulmonary metabolic energy consumption during walking

**Fig. 5:**
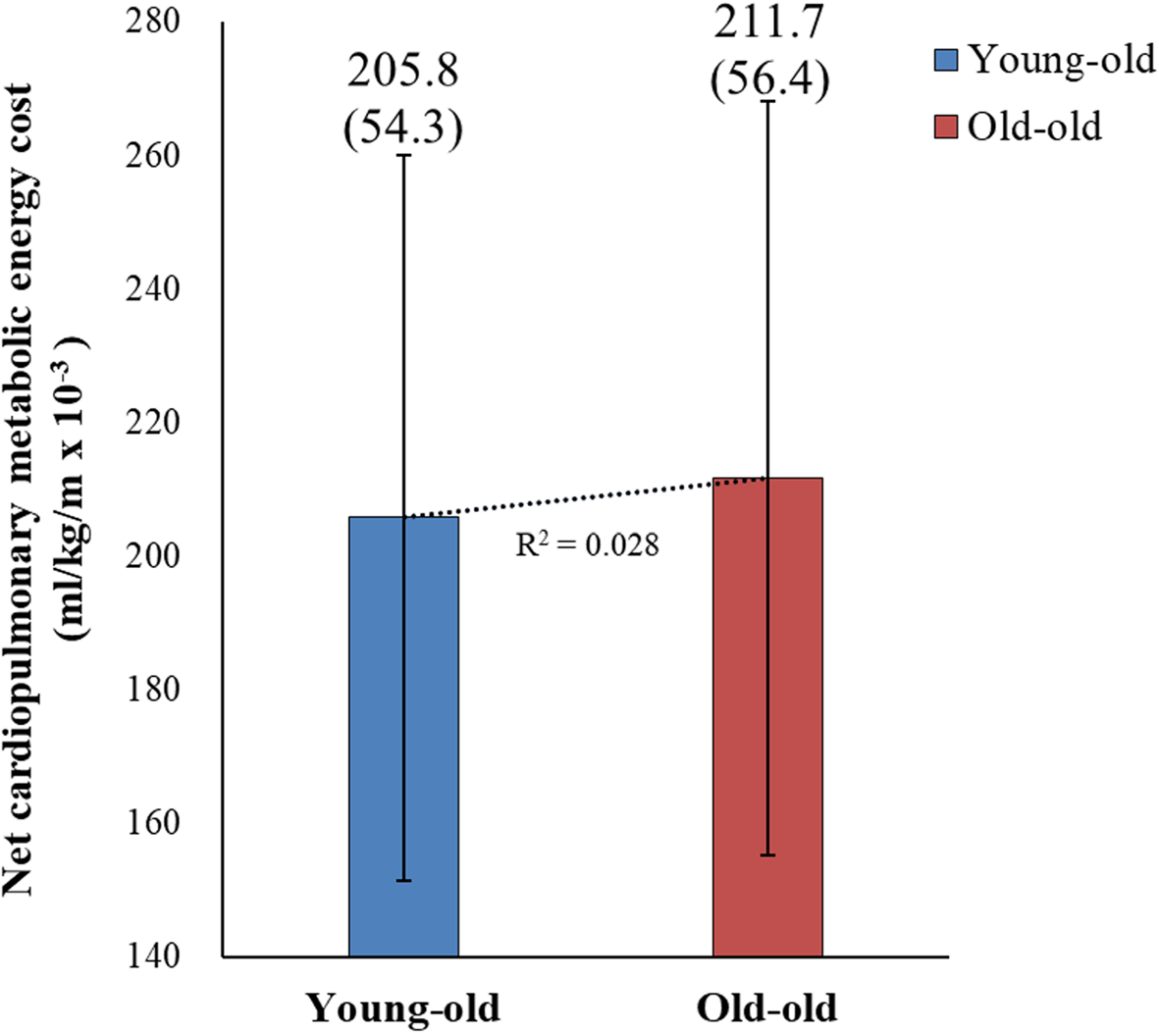
Net cardiopulmonary metabolic energy cost by speed showed a higher net energy cost in the old-old group than the young-old group, but this was without statistical significance

Table 3 shows no significant differences in net VO2, EEm, and MET between the young-old and old-old groups.Preferred treadmill walking speed and distance are presented in Supplementary Table S4. Figure 5 demonstrates that the net cardiopulmonary metabolic energy cost by speed was slightly higher for the old-old group during walking compared to the young-old group, although this difference did not reach statistical significance.

**Table 3:** Differences in metabolic energy consumption between the young-old and old-old groups

	Young-old	Old-old	*P* value
Net VO2 [ml/kg/min]	11.58 (2.57)	11.29 (3.63)	0.721
EEm [kcal/min]	4.73 (1.01)	4.60 (1.16)	0.631
MET	4.62 (0.74)	4.41 (0.91)	0.303

Values are presented as mean (standard deviation)

*Abbreviations*: *Net VO2* net cardiopulmonary metabolic energy cost, *EEm* energy expenditure measurement, *MET* metabolic equivalent

## Discussion

In this study, we investigated differences in gait characteristics and cardiopulmonary metabolic energy consumption during walking between young-old (65–74 years) and old-old (75–84 years) participants using a 3D motion capture system and a wearable cardiopulmonary energy measurement system.

Physical performance according to SPPB score was lower in the old-old group than the young-old group, and the FSST and TUG results were slower in the old-old group than the young-old group. In the mood state and quality of life assessments, the GDS-SF score of the old-old group was higher and the EQ-5D score significantly lower than those in the young-old group. Older people with a slower gait speed move at a slow pace when performing daily tasks such as moving from sitting in a chair to standing and walking. Those changes are caused by a decline in muscle strength, reduced physical exercise, joint pain, and fear of falling [45–48]. Previous research has demonstrated that decreased physical function causes depression to worsen [49], and poorer physical function also causes a decline in social activities, which in turn causes a decrease in quality of life [50]. The results of this study support an impact of decreased physical function in the old-old group based on GDS-SF and EQ-5D scores.

Analysis of spatiotemporal gait parameters showed that the old-old group tended to walk at a slower speed and use shorter step length and stride length than the young-old group. Research suggests that walking speed has a positive correlation with step length and stride length in older adults [51]. Older adults tend to move at slower speeds with shorter strides because of reduced muscle strength and limited ability to control the balance, which affects fall risk [52]. The analysis of spatiotemporal gait parameters in this study revealed that the old-old group walked at a slower speed and used shorter step length and stride length than the young-old group, indicating a higher risk of falls in the older participants. These results suggest various methods such as gait rehabilitation exercises and walking programs be used to improve older people walking ability and reduce the risk of falls to improve quality of life and safety of old adults.

The study's kinematic findings indicated that the old-old group tended to have greater knee flexion upon heel contact than the young-old group. A prior study suggested that a reduced extension angle of the knee joint during initial contact in older adults is linked to weaker quadriceps muscles, knee pain, and slower walking speed [53, 54]. Not extending the knees fully while walking can lead to increased weight load on the knees, which can result in pain and increase the risk of falling [55]. The results suggest that a decrease in the extension angle of the knee joint could potentially serve as an early indication of knee issues associated with aging. The angle of plantarflexion at the ankle usually decreases as people age due to weakened lower limb muscles and reduced power to lift them, leading to slower gait speed [56, 57]. The results of the current study were consistent with previous research, with the old-old group utilizing less plantarflexion in their ankle than the young-old group during the initial swing phase.

Aging reduces the hip extension moment during the loading response phase [58]. In this study, a similar trend was observed, but no significant differences were found between groups. Additionally, the old-old group exhibited significantly lower hip joint maximum flexion moment, which indicates weakness in swinging and kicking the lower limbs to generate forward propulsive force while walking [59]. This is related to decreased absorption force in the knee joint, which results in greater pressure being exerted on the knee joints without effectively absorbing the repelling force when compared to the young-old group [60]. Overall, this means that the joints absorb rather than generate energy at different stages of the gait cycle. This can lead to changes in gait to compensate for joint pain or stiffness with decreased muscle strength, especially in the hip and knee joints, with increased dependence on joint absorption during the preswing phase. These results suggest that eccentric training of the quadriceps muscles might enhance power absorption and alleviate knee joint pain or dysfunction in old-old people.

In cardiopulmonary metabolic energy consumption, despite walking slower than the young-old group, the old-old group used a similar amount of energy in terms of net VO2, EEm, and MET during preferred walking speeds on the treadmill. One possible explanation for the lack of statistically significant difference between the groups is that the average age of the old-old group was 77.74 years, which isn’t much older than 75; another possible reason is that this study recruited very healthy old-old participants.

This study has some limitations. First, although aging affects both gait pattern and muscle activity, this study did not measure muscle activity. In a future study, muscle activation of the lower limbs should be measured alongside kinematic and kinetic data. Second, this study did not calculate rigid body model and COM variables. Therefore, future studies should consider specific variables that describe the essential characteristics of movement. In addition, data should be collected for young adults and the oldest-old population (85 +) to compare age-related gait changes more comprehensively.

## Conclusions

In this study, the old-old group had lower functional assessment scores, increased knee flexion angle, impaired ankle joint motion, and insufficient hip and knee joint kinetic values while walking compared to the young-old group. These results will contribute to the development of personalized intervention plans for older adult individuals of varying age to prevent age-related gait issues like falls.

## Supplementary Information

**Additional file 1: Supplementary Table S1.** Kinematic joint angle differences between the young-old and old-old groups. **Supplementary Table S2.** Kinetic peak joint moment differences between the young-old and old-old groups. **Supplementary Table S3.** Kinetic peak joint power differences between the young-old and old-old groups. **Supplementary Table S4.** Self-selected treadmill walking speed and distance in the young-old and old-old groups. **Supplementally Figure S1.** Peak ground reaction force over a gait cycle did not differ significantly between the young-old and old-old groups. IC: Initial contact (0–2%), LR: Loading response (2–12%), MS: Mid-stance (12–31%), TS: Terminal stance (31–50%), PSw: Pre-swing (50–62%), ISw: Initial swing (62–73%), MSw: Mid-swing (73–87%), TSw: Terminal swing (87–100%).

## Data Availability

The datasets used and/or analyzed during the current study available from the corresponding author on reasonable request.

## Abbreviations

BMI: Body Mass Index
SPPB: Short Physical Performance Battery
FSST: Four-Square Step Test
TUG: Timed Up and Go
K-MBI: Korean version of the Modified Barthel Index
GDS-SF: Geriatric Depression Scale Short Form
K-MMSE: Korean Mini-mental State Examination
EQ-5D: EuroQol-5 Dimensions
FES-K: Fall Efficacy Scale-Korea
