# Supplementary material for: Comparative study of young-old and old-old people using functional evaluation, gait characteristics, and cardiopulmonary metabolic energy consumption
Source: BMC Geriatr. 2023 Jun 29;23:400. doi: 10.1186/s12877-023-04088-6 (PMC10311791; doi:10.1186/s12877-023-04088-6)
Supplement: Supplementary file 1 — Additional file 1: Supplementary Table S1. Kinematic joint angle differences between the young-old and old-old groups. Supplementary Table S2. Kinetic peak joint moment differences between the young-old and old-old groups. Supplementary Table S3. Kinetic peak joint power differences between the young-old and old-old groups. Supplementary Table S4. Self-selected treadmill walking speed and distance in the young-old and old-old groups. Supplementally Figure S1. Peak ground reaction force over a gait cycle did not differ significantly between the young-old and old-old groups. IC: Initial contact (0–2%), LR: Loading response (2–12%), MS: Mid-stance (12–31%), TS: Terminal stance (31–50%), PSw: Pre-swing (50–62%), ISw: Initial swing (62–73%), MSw: Mid-swing (73–87%), TSw: Terminal swing (87–100%). [file 12877_2023_4088_MOESM1_ESM.pdf]

Supplementary material

Supplementary Table S1. Kinematic joint angle differences between the young-old and old-old groups.

Variables	Angle [degrees]		
	Young-old	Old-old	*P* value
Hip			
Initial contact	34.29 (7.82)	34.73 (7.80)	0.825
Loading response	31.82 (7.92)	31.83 (7.53)	0.995
Mid stance	19.56 (8.51)	19.28 (7.97)	0.895
Terminal stance	1.88 (8.56)	1.86 (8.96)	0.994
Pre swing	-3.77 (8.25)	-4.54 (8.70)	0.720
Initial swing	16.54 (8.14)	14.39 (9.52)	0.343
Mid swing	34.84 (7.07)	33.40 (9.23)	0.494
Terminal swing	35.98 (7.39)	36.10 (8.89)	0.955
Knee			
Initial contact	11.73 (4.98)	15.23 (6.42)	**0.019**§
Loading response	18.27 (4.85)	20.56 (6.73)	0.131
Mid stance	20.36 (5.40)	22.04 (6.82)	0.287
Terminal stance	13.96 (5.88)	15.13 (6.08)	0.445
Pre swing	28.55 (6.91)	26.99 (7.03)	0.380
Initial swing	63.33 (5.28)	61.12 (8.41)	0.221
Mid swing	57.34 (7.69)	59.80 (10.08)	0.286
Terminal swing	19.15 (7.20)	24.02 (8.07)	**0.015***
Ankle			
Initial contact	-5.75 (4.01)	-4.50 (4.21)	0.237
Loading response	-8.31 (2.92)	-6.98 (3.99)	0.137
Mid stance	-0.42 (2.73)	0.42 (4.46)	0.373
Terminal stance	8.22 (3.19)	8.78 (4.11)	0.550
Pre swing	5.92 (4.82)	9.63 (4.86)	**0.004****
Initial swing	-9.96 (4.15)	-6.16 (5.57)	**0.003****
Mid swing	-2.86 (3.90)	-2.33 (4.55)	0.621
Terminal swing	-3.08 (3.69)	-1.99 (3.90)	0.265

Values are presented as mean (standard deviation). Initial contact (0–2%), loading response (2–12%), mid-stance (12–31%), terminal stance (31–50%), pre-swing (50–62%), initial swing (62–73%), mid-swing (73–87%), terminal swing (87–100%). Hip flexion (+)/extension (−); knee flexion (+)/extension (−); ankle dorsiflexion -

(+)/plantarflexion (−). *Significant difference between the young-old and old-old groups on paired t-test. *$P < 0.05$, **$P < 0.01$. §Significant difference between the young-old and old-old groups on Mann-Whitney U test. §$P < 0.05$.

Supplementary Table S2. Kinetic peak joint moment differences between the young-old and old-old groups.

Variables	Moment [N·m/kg·m]		
	Young-old	Old-old	*P* value
Hip			
Extension	1.45 (0.67)	1.15 (0.51)	0.053
Flexion	-0.96 (0.38)	-0.77 (0.36)	**0.039***
Knee			
Extension	0.47 (0.27)	0.53 (0.28)	0.426
Flexion	-0.29 (0.13)	-0.25 (0.15)	0.210
Ankle			
Plantarflexion	1.16 (0.31)	1.12 (0.29)	0.629
Dorsiflexion	-0.10 (0.08)	-0.12 (0.12)	0.627

Values are presented as mean (standard deviation). Hip extension (+)/flexion (−); knee extension (+)/flexion (−); ankle plantarflexion (+)/dorsiflexion (−). *Significant difference between the young-old and old-old group on paired t-test. *$P < 0.05$.

Supplementary Table S3. Kinetic peak joint power differences between the young-old and old-old groups.

Variables	Power [W/kg·m]		
	Young-old	Old-old	*P* value
Hip			
1st generation	2.02 (0.93)	1.61 (0.76)	0.064
Absorption	-0.76 (0.50)	-0.61 (0.45)	0.240
2nd generation	1.62 (0.56)	1.49 (0.65)	0.416
Knee			
Generation	-0.83 (0.27)	-0.79 (0.26)	0.646
Absorption	-2.10 (1.06)	-1.60 (0.79)	**0.035***
Ankle			
Generation	-0.88 (0.32)	-0.98 (0.39)	0.321
Absorption	1.73 (0.59)	1.47 (0.44)	0.060

Values are presented as mean (standard deviation). Hip generation (+)/absorption (−); knee generation (+)/absorption (−); ankle generation (+)/absorption (−). *Significant difference between the young-old and old-old group on paired t-test. *$P < 0.05$.

Supplementary Table S4. Preferred treadmill walking speed and distance in the young-old and old-old groups.

	All participants	Young-old	Old-old	*P* value
Treadmill speed [km/h]	3.34 (0.50)	3.48 (0.47)	3.20 (0.51)	**0.025***
Distance	332.00 (57.01)	346.90 (58.13)	318.06 (53.13)	**0.002****

Values are presented as mean (standard deviation). *Significant difference between the young-old and old-old group on paired t-test. *$P < 0.05$, **$P < 0.01$.

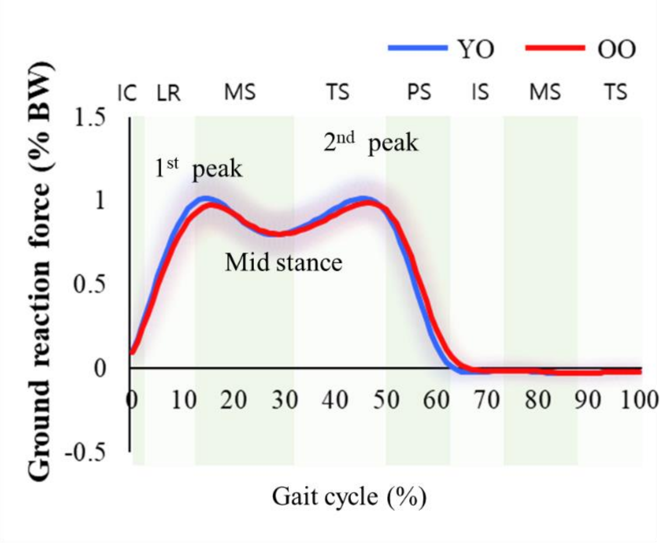

Supplementally Figure S1. Peak ground reaction force over a gait cycle did not differ significantly between the young-old and old-old groups. IC: Initial contact (0–2%), LR: Loading response (2–12%), MS: Mid-stance (12–31%), TS: Terminal stance (31–50%), PSw: Pre-swing (50–62%), ISw: Initial swing (62–73%), MSw: Mid-swing (73–87%), TSw: Terminal swing (87–100%).
